# What Can You Do With a Bachelor's in Public Health? A Case Study of Graduate Outcomes From the University of Hawai‘i

**DOI:** 10.3389/fpubh.2021.661629

**Published:** 2021-08-09

**Authors:** Denise C. Nelson-Hurwitz, Michelle Tagorda, Lisa Kehl, Uday Patil

**Affiliations:** Office of Public Health Studies, Thompson School of Social Work & Public Health, University of Hawai‘i at Mānoa, Honolulu, HI, United States

**Keywords:** public health education, bachelors of public health, undergraduate public health, workforce development, public health workforce, undergraduate public health education, workforce shortage

## Abstract

Since the development of a Bachelor of Arts in public health, a common question among prospective students, faculty, and community members has been asked: “What will students do after graduation?” While national data on graduates are abundant, community inquiries sought more detailed profiles of local graduates. To address this need, data on 224 graduates of the Bachelors of Arts in Public Health (BAPH) degree at the University of Hawai‘i at Mānoa were collected through alumni outreach efforts and professional online profile searches. Data were compiled into a summary presentation and program “resume”. Findings indicated roughly 30% of BAPH graduates pursued graduate-level education and 43% were employed within a year of graduation. Employment in local NGOs and healthcare organizations was common, while bureaucratic challenges limited hiring at public agencies. A review of job titles suggested graduates were employed in program coordination or support staffing. Local program profiles, such as this one, complement national employment data. Moreover, these efforts may foster interest among prospective students and support from faculty, administrators, and employers.

## Introduction

Public health has never been more important than during this troubling time of both pandemic and health infodemic. Mass media exposure to public health professions has fostered community interest and created prospective students.

The State of Hawai‘i has been short of healthcare workers perennially, particularly those trained in public health. An estimated 2200 non-physician healthcare positions were open between 2018 and 2019 (1, 2). Moreover, the demand is anticipated to increase over the next 5 years (2) as the state attempts to recover from the COVID-19 pandemic. Other potential drivers of the demand include family and financial considerations (e.g., high cost of living, non-competitive salaries, and high debt burden). Challenges in the educational pipeline (e.g., retiring workforce and insufficient local training) have been noted consistently (3, 4). The need for both specialist (e.g., MPH) and generalist (BA) graduates is apparent as public health is increasingly promoted in schools, communities, and other places aside from the clinical setting.

As only 17% of public health professionals are formally trained in public health nationwide (5, 6) undergraduate public health programs are essential to train and support an evolving labor force (6–8). The historic Institute of Medicine report from 2003 calling for undergraduate education set the tone, and guideposts and practical tools for such alignment between public health needs and undergraduate education followed shortly thereafter (9) as formulated systematically in ASPPH's 2011 learning outcomes model and the program template from in 2011 (10). Frameworks have also been developed by AAC&U and CEPH (11). Now, local colleges and universities are reevaluating this challenge in the midst of a global pandemic (8). However, the effectiveness of these efforts has not been studied well, as data on student outcomes is nascent and crude (12, 13). The degree itself is no older than 50 years (14, 15). Even though 271 institutions conferred undergraduate public health degrees in 2016, only one-third of these programs existed in 2003 (8).

In a recent review of 33 student outcome reports, Krasna et al. mainly found individual case studies of program satisfaction with little systematic reporting and difficulty comparing outcomes between schools. The heterogeneity among curriculum among a large number of program types (i.e., BA, BS, BA/BS, and BAPH/BSPH/BPH) with public health majors do not allow for easy cross-comparison of outcomes, as induced from the review by Holsinger et al. (6).

Detailed program-specific analyzes of graduate outcomes are rare (13). Previously, Plepys et al. (16) found 57–65% of BAPH graduates were employed as a first-destination graduate outcome. A broad survey of post-graduate outcomes came from the ASPPH report on 1,300 graduates: 75% were employed and 12% were enrolled in graduate or professional school and called for further detailed inquiries into post-graduation outcomes (8, 17). Tulane University School of Public Health and Tropical Medicine found 66–82% of bachelor's graduates continued with further education; those looking for employment found jobs largely outside of government health agencies (18). But these findings are only applicable to a minor proportion of public health graduates, and none could reasonably be applied to the unique context of the undergraduate public health program at the University of Hawai'i.

A global review of public health student outcomes yielded much information into the outcomes of master's and doctorate graduates but little regarding those earning bachelor's degrees. A detailed investigation by Dlungwane and Knight (19) focused on the diverse employment of MPH graduates in South Africa, while Li and Awofeso (20) had a similar query of MPH graduates from Australia. Watts et al. conducted two studies finding roughly 40% of public health job listings in Australia and New Zealand accepted bachelor's holders, (but with not enough specificity to identify the jobs made available to BAPH graduates) (21), and indicated a nearly a 3-fold increase in BAPH graduates since 2001 in Australia, (but nothing on the vocational or educational outcomes of graduates) (22). Finally, the Swiss School of Public Health conducted a survey of the national public health workforce and identified roughly 30% of respondents to hold a bachelor's degree or less (23), suggesting bachelor's graduates are represented and needed in the workforce. However, the findings are not specific enough to identify the first-destination outcomes or later trajectories of public health bachelor's degree earners. The global lack of outcome data may limit the true evaluation and subsequent expansion of BAPH programs.

### Case

The University of Hawai‘i at Mānoa (UHM) is a land-grant university, public educational center intended to provide high-quality education in service to the local and indigenous community. Besides traditional health sciences academic programs, UHM provides practical services and training to meet local vocational needs. The Office of Public Health Studies (OPHS) graduate program served as the primary educational pipeline for the local public health workforce since 1962, granting both masters and doctoral degrees (24).

As the public health response evolved (25) and the workforce approached retirement, OPHS recruited younger faculty with the offering of a Bachelor of Arts in Public Health (BAPH) in January 2014 (26). The BAPH at the University of Hawai‘i at Mānoa graduated its first students in December 2015. Between December 2015 and May 2020, 224 degrees have been awarded. Program development mirrored national trends of substantial growth in undergraduate public health degree offerings (8, 27).

Multiple stakeholders collaborated in the development of the BAPH and in the process raised a plethora of difficult but necessary questions (28). All partners shared a common inquiry: what will students do after graduation? Due to the nascency of the public health bachelor's program landscape, limited data on alumni employment, particularly first-destination employment, were available. Current research findings painted a national, not local, picture (8, 29).

Regardless, BAPH graduates filled local health agencies and employers requested graduates to fit specific job titles and sites. In time, OPHS faculty collected this data and much more from BAPH graduates in order to address community and industry inquiries methodically and meet Council for Education in Public Health (CEPH) accreditation standards. Here, we share collected BAPH graduation outcomes with implications for community dissemination.

## Methods

Current education and job information was collected, including field of study/employment, to identify continuity within the public health sector. One year after commencement, faculty and staff inquired into every graduate's outcomes. First, a Student Support Specialist noted students on the commencement roster who transitioned into graduate programs within OPHS (e.g., MPH, MS in Epidemiology). The same staff member utilized the MyUH enrollment management system to capture outcome data on other continuing students in closely affiliated health sciences programs (e.g., MSW, RN, MD, PharmD). While a notable portion of the graduating class continued studying at UHM, other graduates were harder to survey.

Then, the Specialist transferred any results from the Undergraduate Alumni Update Form, which is regularly promoted by faculty whenever graduates contact the program or faculty. The form polls current positions, as well as accomplishments and achievements. Often, faculty and staff interact regularly with recent graduates working with partnered social service organizations and affiliated clinical settings; any student outcomes gleaned from these personnel were relayed to the Specialist, who then entered the information into the outcomes database.

Next, the Specialist searched the LinkedIn social networking site for publicly available data regarding current employment or enrollment of the remaining graduates and combined these findings from public updates on Facebook and Instagram. A targeted email to undergraduate faculty requesting new contact information or outcomes findings for recent graduates was sent also. This procedure determined the outcomes for many remaining undetermined cases. Many instances of graduate-initiated contact with faculty occurs annually due to the close-knit community context, limited class sizes, and overlapping advising and teaching roles of many staff. When letters of recommendation or reference requests are made, faculty consistently asked about post-graduation schooling and employment outcomes. The aggregate findings in the student outcomes database were confirmed and verified by the Undergraduate Chair.

While a standardized survey to determine graduate outcomes was developed, the dissemination of a mass survey was not ideal for three reasons: (a) logistical difficulties caused by the pivot to distance learning; (b) cultural considerations of sharing personal and professional updates; and (c) a need for a concise, informal questionnaire to complement regular, in-depth surveys for eventual reporting to CEPH, the University of Hawai‘i System, and the Office of Public Health Studies' Community Advisory Panel. Therefore, OPHS chose to collect data individually, when possible. The data collection procedure served to compile all outcome data into a singular database for analysis.

## Results

From program inception to the conclusion of the Spring 2020 semester, 224 students had graduated with a BAPH degree from the University of Hawai‘i at Mānoa. The average graduating class size is 44 with a range of 32 to 56 graduates per academic year. Of the 224 graduates, 67 (29.9%) were pursuing continued education, 98 (43.8%) were employed, 8 (3.6%) were seeking employment, 4 (1.8%) were not seeking employment), 4 (1.8%) were Peace Corps Volunteers, and 43 (19.2%) were undetermined due to lack of response. Of the 67 graduates continuing their education, 29 (43.3%) were pursuing graduate degrees in public health, 17 (25.4%) were enrolled in nursing programs, either at the bachelor's or master's degree level, 5 (7.5%) were in medical school, and 11 (16.4%) were in allied health, including social work, nutrition, chiropractic, or pharmacy programs. It is essential to note a limitation of this survey methodology—the risk of selection bias is high, as non-respondents may have a higher rate of unemployment, underemployment, or no pursuit of further education.

Of the 98 graduates reporting employment, 63 (64.3%) were employed in health-related positions. Employment sites included local public health non-profit agencies, residential living facilities, clinical care settings, or governmental public health branches, in Hawai‘i and throughout the Pacific. Only one graduate was employed at the local department of health where challenges exist with hiring procedures, including required years of work experience, and what opportunities may be considered toward this experience. Similar challenges are reported nationally (30). Other employed graduates worked in educational settings (8 graduates), insurance agencies (4 graduates), or reported active military service (4 graduates). Common job titles included administrative assistant; program specialist; and program, health, or wellness coordinator, suggesting BAPH graduates were engaged primarily in entry-level positions.

### Dissemination and Communication

**Figure 1:**
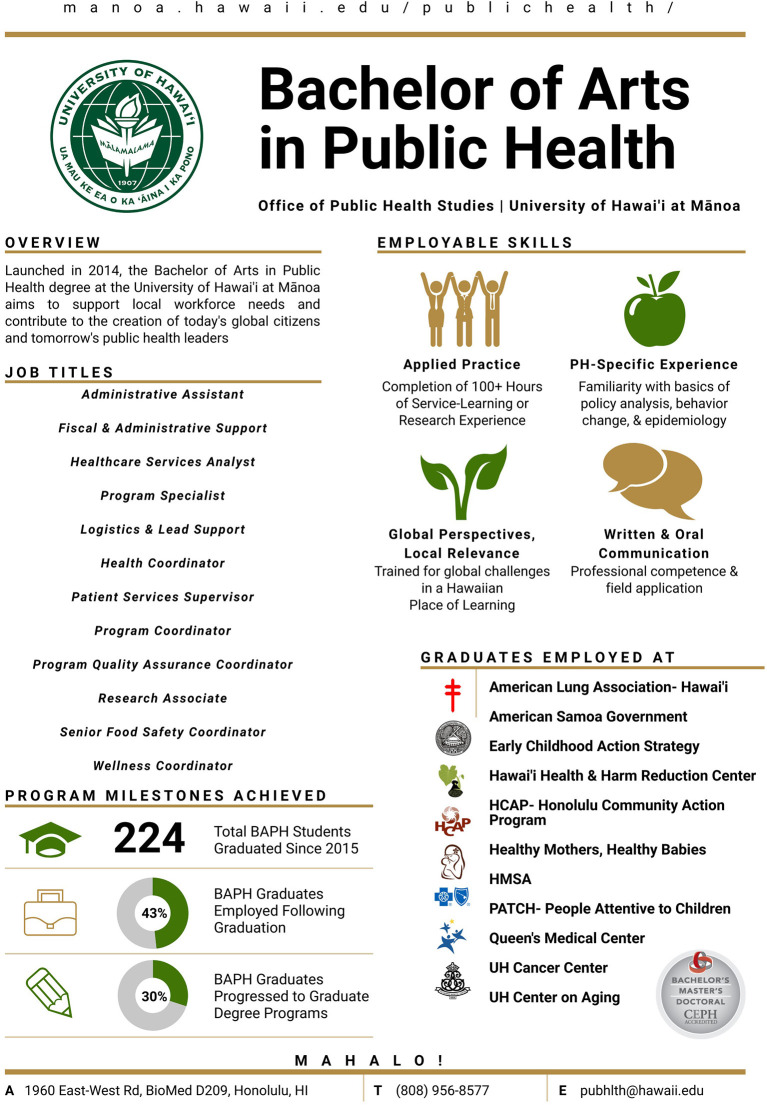
BAPH Graduate Summary Outcomes Communication Tool.

To facilitate communication of alumni data to the OPHS' Community Advisory Panel, as well as to respond to inquiries from community members and prospective students, alumni data was summarized in the format of a resume for the BAPH program. The program resume features summary information regarding the program, including key marketable skills, and includes sample position titles of current alumni, as well as a sampling of local and national agencies employing recent BAPH graduates. This communication tool (Figure 1) has since been utilized at community outreach and student recruitment events and has anecdotally received very positive feedback.

## Discussion

Maintenance and growth of the healthcare workforce are critical and of increasing significance given both an aging workforce looking toward retirement (4) and the impacts of COVID-19. In areas previously identified as workforce shortage areas, meeting these local workforce needs is increasingly urgent. As Hawai'i has difficulty retaining outside talent to fill open positions due to its geographic isolation and unique cultural context, it is beholden on local, public universities to develop and promote educational opportunities to address such needs.

Employment data collected clearly shows the BAPH program at OPHS is filling public health employment opportunities, especially in entry-level public health positions, both locally and nationally. BAPH graduates are more likely to enter the job market immediately after graduation compared to graduates elsewhere due to the higher cost of living and larger proportion of jobs that do not require graduate or professional degrees; this finding supports the assertion by Resnick et al. (8). Compared to data from the continental U.S. (7, 16), employment in healthcare was common, though a greater portion of OPHS graduates is employed in local non-profits and less with local government. Identifying the wide variety of employing organizations and specific job responsibilities in the survey not only informed the unique public health landscape (i.e., “public health in action”) in Hawai‘i, but also possibilities for service learning of current students.

For alumni, entry-level employment has allowed those with a BAPH degree to both build meaningful careers in public health and gain valuable work experience in anticipation of graduate program application. Only through the methodical collection of detailed graduate outcome data, such as specific job titles and descriptions, could these determinations be made. For alumni looking to enter graduate degree programs immediately upon graduation, the BAPH program provides a strong educational foundation and a pipeline for graduate-level education. Consequently, OPHS master's degrees have appreciated access to an engaged and well-prepared student body from which to recruit.

### Implications

From a programmatic perspective, regularly reviewing alumni data serves both program evaluation purposes and helps support proposed revisions to curriculum and student support. In evaluation, alumni data help faculty identify current, and evolving, training and skills needed for highly marketable candidates. It further allows the BAPH program to remain sensitive to relevant community needs. It is especially critical in Hawai‘i and the Pacific to continuously gauge local and regional community needs, due to unique challenges of workforce recruitment and retention (3, 4). These data, and the developed communication tool, were further helpful in garnering support for the program, and later programmatic changes, among faculty, university administration, and community employers. Among prospective students, this has also been helpful in providing a perspective on the diversity of local careers available to BAPH graduates.

While additional analyzes and continuous data collection are necessary, profiles of local public health degree program graduates may be supportive in complementing national graduation data, and further communicating the benefit of local alumni to address community workforce needs.

## Data Availability Statement

The raw data supporting the conclusions of this article will be made available by the authors, without undue reservation.

## Author Contributions

DN-H and MT contributed conception and design of the project and data collection. DN-H wrote the first draft of the manuscript. All authors contributed to data analysis, wrote sections of the manuscript, contributed to manuscript revision, read, and approved the submitted version.

## Conflict of Interest

The authors declare that the research was conducted in the absence of any commercial or financial relationships that could be construed as a potential conflict of interest.

## Publisher's Note

All claims expressed in this article are solely those of the authors and do not necessarily represent those of their affiliated organizations, or those of the publisher, the editors and the reviewers. Any product that may be evaluated in this article, or claim that may be made by its manufacturer, is not guaranteed or endorsed by the publisher.
